# Evolutionary analysis of six chloroplast genomes from three *Persea americana* ecological races: Insights into sequence divergences and phylogenetic relationships

**DOI:** 10.1371/journal.pone.0221827

**Published:** 2019-09-18

**Authors:** Yu Ge, Xiangshu Dong, Bin Wu, Nan Wang, Di Chen, Haihong Chen, Minghong Zou, Zining Xu, Lin Tan, Rulin Zhan

**Affiliations:** 1 Haikou Experimental Station, Chinese Academy of Tropical Agricultural Sciences, Haikou, China; 2 College of Agriculture, Yunnan University, Yunnan, China; 3 College of Agriculture, Guangxi Vocational and Technical College, Nanning, China; 4 South Subtropical Crops Research Institute, Chinese Academy of Tropical Agricultural Sciences, Zhanjiang, China; National Cheng Kung University, TAIWAN

## Abstract

Chloroplasts significantly influence species phylogenies because of their maternal inheritance and the moderate evolutionary rate of their genomes. Avocado, which is a member of the family Lauraceae, has received considerable attention from botanists, likely because of its position as a basal angiosperm. However, there is relatively little avocado genomic information currently available. In this study, six complete avocado chloroplast genomes from three ecological races were assembled to examine the sequence diversity among the three avocado ecological races. A comparative genomic analysis revealed that 515 simple sequence repeat loci and 176 repeats belonging to four other types were polymorphic across the six chloroplast genomes. Three highly variable regions (*trnC-GCA-petN*, *petN-psbM*, and *petA-psbJ*) were identified as highly informative markers. A phylogenetic analysis based on 79 common protein-coding genes indicated that the six examined avocado accessions from three ecological races form a monophyletic clade. The other three genera belonging to the *Persea* group clustered to form a sister clade with a high bootstrap value. These chloroplast genomes provide important genetic information for future attempts at identifying avocado races and for the related biological research.

## Introduction

Avocado (*Persea americana* Mill.) is one of the most economically important subtropical/tropical fruit crops worldwide. It is a member of the family Lauraceae of the order Laurales, which is a large pantropical family comprising about 50 genera and 2,500–3,000 species of mostly trees and some shrubs [1]. According to Chanderbali [2], Laurales species (avocado and relatives) form a key clade, the magnoliids, which includes most basal angiosperms according to the generally recognized angiosperm phylogenetic relationships. Additionally, avocado is now an established genetic model plant species for illustrating angiosperm evolution [2,3]. Taxonomic treatments differ considerably in terms of the circumscription and defining of infraspecific avocado entities [4–7]. Moreover, researchers have long considered that geographical isolation has likely resulted in the following three ecological races of avocado: Mexican (*P*. *americana* var. *drymifolia*), Guatemalan (*P*. *americana* var. *guatemalensis*), and West Indian (*P*. *americana* var. *americana*) [1].

Distinct differences in race are primarily associated with ecological preferences and fruit characteristics [1]. The Mexican race adapted to a Mediterranean climate, and generally produces cold-tolerant and early maturing fruit with a thin, usually purplish-black skin. In contrast, the Guatemalan race is slightly cold-tolerant because it originated in a tropical highland climate, and its fruit has a thick and tough skin, which remains green until the fruit matures. The West Indian race adapted to humid tropical lowland conditions, making it very cold-sensitive, and its fruit has a thin and yellowish green skin, with higher sugar and lower oil contents than the fruits of the other two races [1,8,9]. Guatemalan cultivars, or their hybrids with Mexican accessions, generally produce high-quality fruit, and dominate the germplasm of the ‘subtropical’ avocado cultivars grown and traded worldwide. The Mexican race usually does not produce high-quality fruit, but it has contributed genes for early maturity and cold tolerance. The fruit of West Indian types, or their hybrids with Guatemalan accessions, have relatively low oil contents, but high sugar contents, and dominate the germplasm of the ‘tropical and semi-tropical’ avocado cultivars worldwide [1].

Previous phylogenetic studies have typically revealed diffuse boundaries among the three avocado ecological races. Studies based on morphological characteristics [4] and various molecular markers [10,11] indicated that Guatemalan samples are clustered with West Indian samples. However, other studies involving molecular markers distinguished between the three ecological races [9,12–14]. Therefore, reliable and cost-effective methods are needed to authenticate these three ecological races. There are many advantages to using nuclear and chloroplast genomes and transcriptomes to characterize phylogeny and evolutionary history [15,16]. Generally, genome-scale datasets can be used to illuminate phylogenetic relationships among closely related species [17]. Our previous research on the phylogenetic relationships of the three avocado ecological races based on specific length amplified fragment sequencing [18], transcriptomes examined according to single nucleotide polymorphisms [18], and transcriptomes investigated based on differentially expressed genes [19] revealed that the Mexican and Guatemalan races are more closely related to each other than to the West Indian race. Specific characteristics of chloroplast genome sequences (e.g., uniparental inheritance, low recombination, and low nucleotide substitution rates) make these sequences relevant for resolving the interspecific or intraspecific boundary, and are particularly useful for phylogenetic studies involving diverse species, including *Lonicera* [20], Nymphaeaceae [21], *Ziziphus* [22], *Capsicum* [23], *Rosa* [24], and *Cruciata* [25] species. However, chloroplast genomes have not been used for elucidating the phylogenetic relationships among the three avocado ecological races.

In this study, we obtained the whole chloroplast genomes of six avocado accessions from three ecological races to clarify the structural variations and perform a comparative analysis. The specific objectives of this study were: (1) to completely sequence the chloroplast genomes of six avocado accessions from three ecological races; (2) to characterize the newly generated chloroplast genomes and examine codon usage and repeat sequences; (3) to examine the mutation hotspots as potential molecular genetic markers for further phylogenetic studies; and (4) expound the phylogenetic relationships among three avocado ecological races and delimit the phylogenetic relationships within the *Persea* group, which is a subset of the family Lauraceae.

## Materials and methods

### Ethics statement

In this study, no specific permits were required for the described field studies. The study is not privately-owned or protected in anyway. The field studies did not involve endangered or protected species.

### Sampling and sequencing

Fresh leaves were collected from six avocado accessions [Walter Hole (Mexican race), Duke 7 (Mexican race), Nabal (Guatemalan race), Reed (Guatemalan race), Pollock (West Indian race), and Simmonds (West Indian race)] at the South Subtropical Crops Research Institute, Chinese Academy of Tropical Agricultural Sciences (Zhanjiang, Guangdong, China; latitude: 21°16′ N, longitude: 110°22′ E, and altitude: 30 m above sea level) and at the Guangxi Vocational and Technical College (Nanning, Guangxi, China; latitude: 22°29′ N, longitude: 108°11 E′, and altitude: 79 m above sea level). The collected leaves were immediately dried with silica gel prior to DNA extraction. These six avocado accessions were genotyped as previously described [18] to validate their clonal race. Total genomic DNA was isolated from approximately 5 g silica-dried leaf tissue with the DNeasy Plant Mini Kit (Qiagen, Germany). Approximately 5 μg purified genomic DNA was used to construct paired-end libraries with 400-bp inserts for the subsequent sequencing with an Illumina HiSeq 2500 platform. The quality of the raw sequence reads was assessed with FastQC (version 0.11.2), after which ambiguous and low-quality reads were discarded. The following eight published chloroplast genome sequences were downloaded for a comparison: *Cinnamomum micranthum* (KR014245), *Machilus yunnanensis* (KT348516), *Machilus balansae* (KT348517), *Phoebe omeiensis* (KX437772), *Phoebe sheareri* (KX437773), *Alseodaphne semecarpifolia* (MG407595), *Alseodaphne gracilis* (MG407593), and *Alseodaphne huanglianshanensis* (MG407594).

### Chloroplast genome assembly and annotation

Filtered paired-end reads were first mapped to the *P*. *americana* (KX437771) chloroplast genome with the default parameters of Bowtie (2–2.2.9) [26]. The matched reads were *de novo* assembled with A5-MiSeq (version 20150522 1.2.10) [27] and SPAdes (version 3.9.0) [28]. All contigs were checked against the *P*. *americana* (KX437771) reference genome with MUMmer (version 3.1) [29], and the aligned contigs were oriented according to the reference genome. The results helped determine the relationships between the contig locations, and were useful for filling the gaps between contigs. Finally, the assembled chloroplast genomes were calibrated with Pilon (version 1.18) [30] and annotated with the Dual Organellar GenoMe Annotator [31] and the *P*. *americana* (KX437771) reference genome sequence. The initial annotations were manually verified based on the available information for other closely related species. ARAGORN (version 1.2.38) [32] was used to confirm the tRNAs, whereas GeSeq (version 1) was used for predicting rRNAs [33]. The tRNAs and rRNAs were calibrated based on the *P*. *americana* (KX437771) reference genome. Finally, circular gene maps for each chloroplast genome were drawn with OGDraw (version 1.2) [34]. The fully annotated chloroplast genomes were deposited in the GenBank database (accession numbers are listed in Table 1).

**Table 1:** Summary of six complete chloroplast genomes from three avocado ecological races.

Ecological race	Mexican race		Guatemalan race		West Indian race	
Accession	Walter Hole	Duke 7	Nabal	Reed	Pollock	Simmonds
GenBank accession number	MK959368	MK959371	MK959367	MK959366	MK959369	MK959370
Genome size (bp)	152,597	152,749	152,763	152,733	152,728	152,725
Large single copy (LSC) length (bp)	100,182	93,815	93,845	93,798	93,579	94,107
Small single copy (SSC) length (bp)	18,807	18,830	18,814	18,829	18,775	18,828
Inverted repeat (IR) length (bp)	16804	20052	20052	20053	20187	19895
Number of genes	113	113	113	113	113	113
Number of protein-coding genes (duplicated in IR)	79 (4)	79 (4)	79 (4)	79 (4)	79 (4)	79 (4)
Number of tRNA genes (duplicated in IR)	30(10)	30(10)	30(10)	30(10)	30(10)	30(10)
Number of rRNA genes (duplicated in IR)	4 (4)	4 (4)	4 (4)	4 (4)	4 (4)	4 (4)
Number of genes with one intron (two introns)	21 (2)	21 (2)	21 (2)	21 (2)	21 (2)	21 (2)
Proportion of coding to noncoding regions (%)	44.62	44.61	44.59	44.60	44.47	44.59
Total number of pseudogenes	2	2	2	2	2	2
Average gene density (genes/kb)	0.87	0.86	0.86	0.86	0.86	0.86
GC content (%)	39.13	39.12	39.11	39.10	39.09	39.10
GC content in LSC (%)	37.94	37.88	37.87	37.87	37.84	37.85
GC content in IR (%)	45.57	44.46	44.45	44.44	44.40	44.54
GC content in SSC (%)	33.96	33.95	33.92	33.91	33.9	33.91

### Codon usage, repeat sequence analysis, genome structural analysis, and genome comparison

The amino acid and codon usage was investigated with CodonW software [35]. Additionally, MISA [36] was used to search for SSRs, with the following settings: 10 for mono-, 5 for di-, 4 for tri-, and 3 for tetra-, penta-, and hexa-nucleotide repeat motifs. The REPuter program [37] was used to establish the size and location of repeat sequences, including the complementary, forward, palindromic, and reverse repeat units in the six avocado chloroplast genomes. The lower limit for the repeat size was set as 30 bp, with a repeat identity of 90% and a Hamming distance of 3. The six complete avocado chloroplast genomes were compared and visualized with mVISTA [38]. The nucleotide variability (average pairwise divergence) among the six avocado chloroplast genomes was calculated with DnaSP (version 5.10) [39], with the following settings: step size: 200 bp and window length: 800 bp.

#### AMOVA analysis

An analysis of molecular variance (AMOVA) based on 484 SNPs and 10,000 permutations were carried out using Arlequin v3.11 [40].

#### Phylogenetic analysis

Phylogenetic analyses were conducted with the aligned data for six new avocado (*P*. *americana*) chloroplast genomes (from this study) as well as the complete chloroplast genomes from the following species: *C*. *micranthum* (KR014245), *M*. *yunnanensis* (KT348516), *M*. *balansae* (KT348517), *P*. *omeiensis* (KX437772), *P*. *sheareri* (KX437773), *A*. *semecarpifolia* (MG407595), *A*. *gracilis*, (MG407593), and *A*. *huanglianshanensis* (MG407594). Phylogenetic trees were constructed with the MP, ML, and BI methods. First, 79 protein-coding genes present in all genome sequences were extracted and aligned with CLUSTAL X [41]. The ML and MP analyses were conducted using MEGA X [42], with 1,000 bootstrap replicates. The BI analyses were completed using MrBayes (version 3.2.6) [43], with the following settings: 1,000,000 simulations with the Monte Carlo algorithm and sampling after every 1,000 simulations. The first 25% of all trees were discarded, and the remaining 75% was used to construct the majority-rule consensus tree, with posterior probabilities for each node.

## Results

### Complete chloroplast genome features

**Fig 1:**
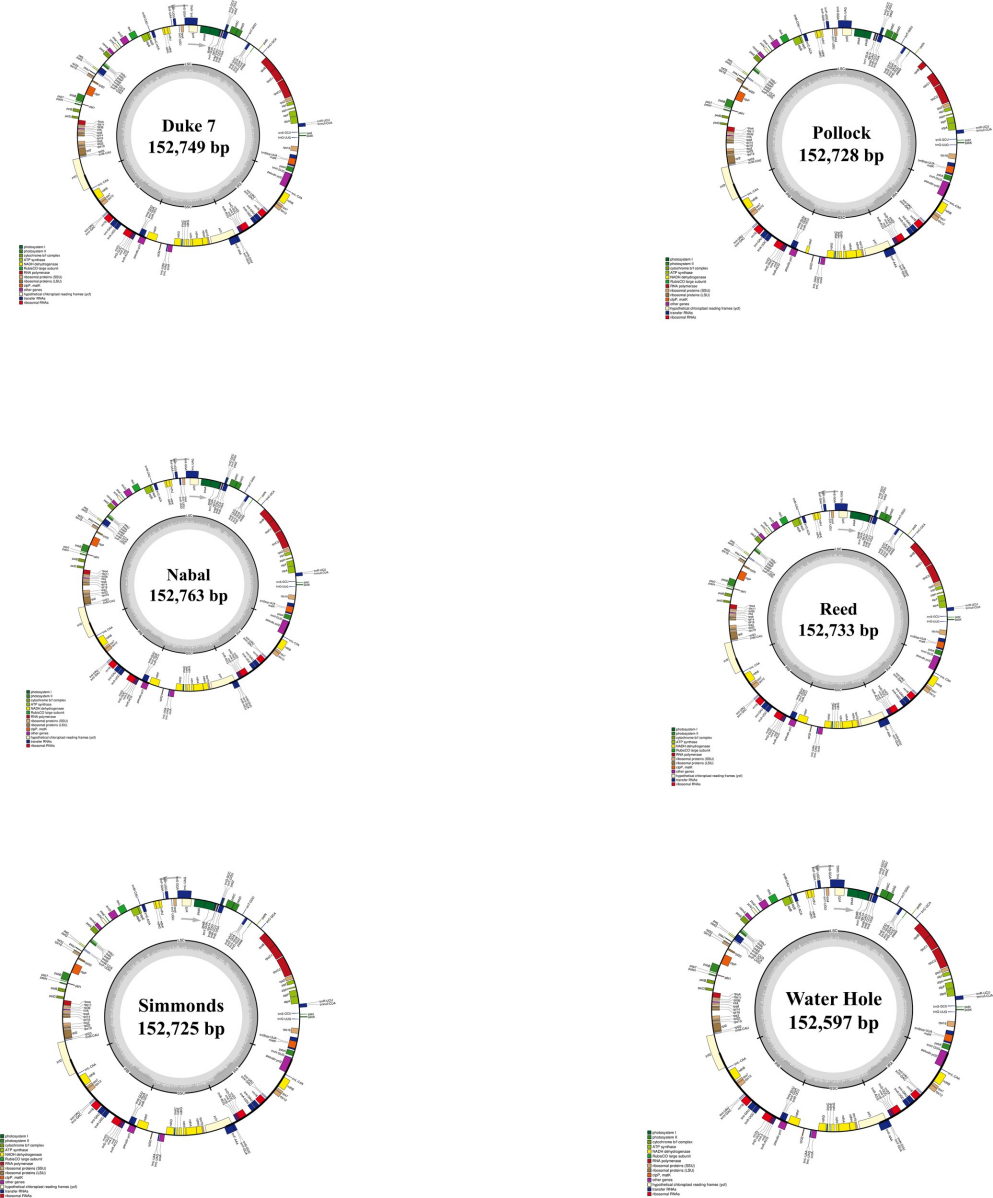
Circular gene maps of six chloroplast genomes from three avocado ecological races. Genes drawn outside the outer rim are transcribed counterclockwise, whereas those inside the outer rim are transcribed clockwise. The colored bars indicate various functional groups, and the area shaded in gray in the inner circle represents the GC level of the corresponding genes. LSC, large single copy; SSC, small single copy; IR, inverted repeat.

The avocado chloroplast genomes of Walter Hole (Mexican race), Duke 7 (Mexican race), Nabal (Guatemalan race), Reed (Guatemalan race), Pollock (West Indian race), and Simmonds (West Indian race) were sequenced, generating approximately 1.09, 1.24, 1.48, 1.53, 1.49, and 1.50 Gb of paired-end reads, respectively. The 125-bp raw reads were trimmed to obtain the clean reads for assembly. After filtering for quality, 4,385,300, 5,226,094, 6,521,846, 6,693,404, 6,559,258, and 6,667,702 high-quality clean reads were generated for Walter Hole, Duke 7, Nabal, Reed, Pollock, and Simmonds, respectively. The six chloroplast genomes were acquired based on *de novo* and reference sequence assembly. The genomes ranged from 152,597 bp (Walter Hole) to 152,763 bp (Nabal) in length and the depth of coverage for the chloroplast genome contigs ranged from 3717× (Walter Hole) to 5693× (Simmonds) (Fig 1, Table 1). A structural analysis revealed that the genomes formed a typical quadripartite structure containing a large single copy (LSC) region (93,579–100,182 bp), a small single copy (SSC) region (18,775–18,830 bp), and a pair of inverted repeat (IR) regions (16,804–20,187 bp) (Table 1). There were no significant differences in chloroplast genome length and structure among the three avocado ecological races. The overall guanine and cytosine (GC) content was similar in the six chloroplast genomes (approximately 39.11%) (Table 1). Additionally, the GC content was asymmetrically distributed across the chloroplast genome, with the highest content detected in the IR regions (44.64%), followed by the LSC (37.88%) and SSC (33.93%) regions. The generated six chloroplast genomes from three avocado ecological races were deposited in the GenBank database (accession number MK959366-MK959371).

**Fig 2:**
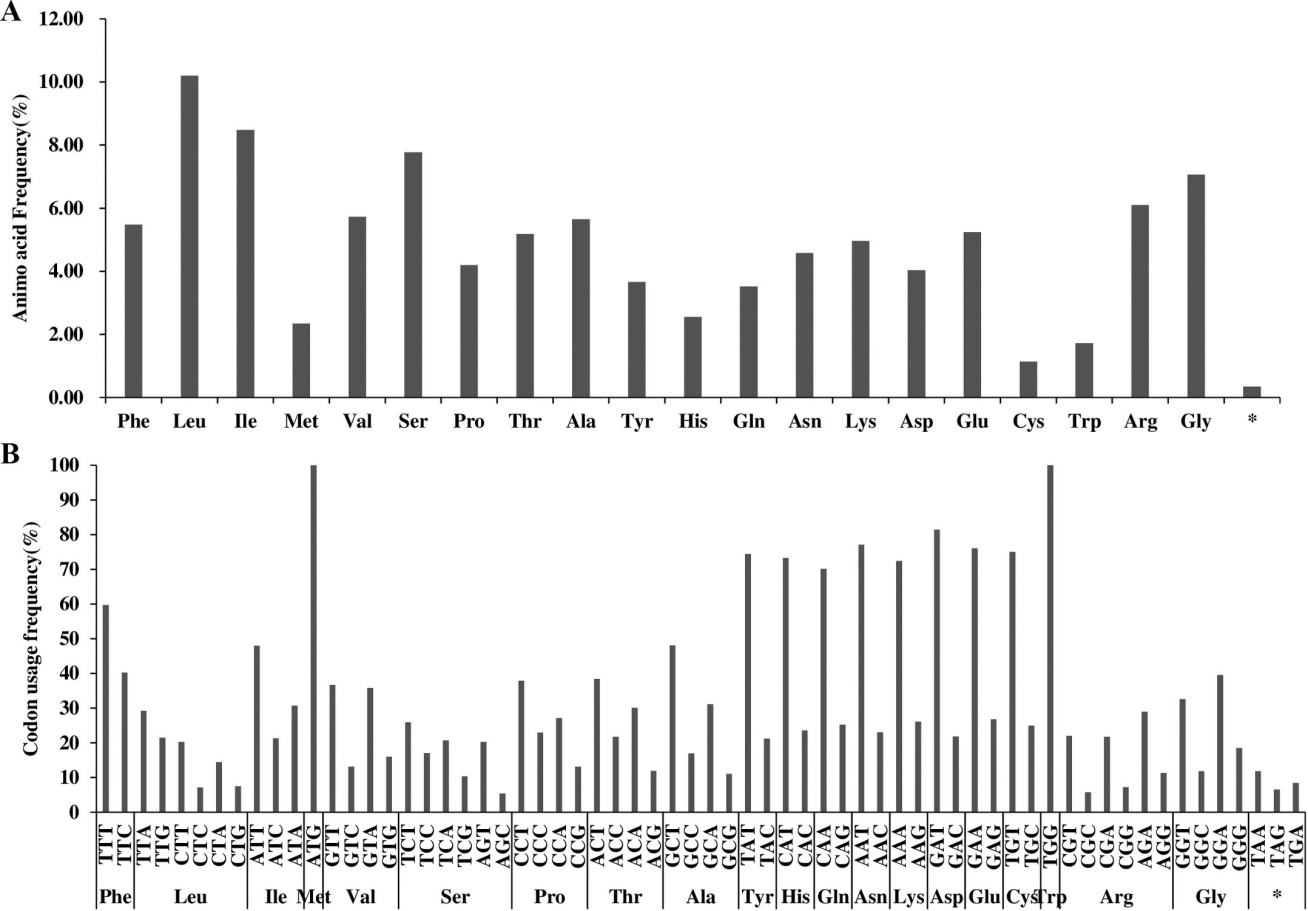
**Amino acid (A) and codon usage (B) frequencies of the protein-coding sequences in six avocado chloroplast genomes.** *: Termination codon.

The six analyzed chloroplast genomes contained 113 unique genes arranged in the same order, including 79 protein-coding genes, 30 tRNA genes, and 4 rRNA genes, 18 of which were duplicated in the IR regions (Table 1, S1 Table). Two sequences (*ycf1* and *ycf2*), which were present in all six chloroplast genomes, were identified as pseudogenes (S1 Table). An analysis of the codon and encoded amino acid frequencies of the protein-coding sequences in the six chloroplast genomes (Fig 2) revealed the presence of 61 codons encoding 20 amino acids. The protein-coding genes encoded 22,635 amino acids in Walter Hole, 22,711 amino acids in Duke 7, 22,664 amino acids in Nabal, 22,704 amino acids in Reed, 21,136 amino acids in Pollock, and 22,701 amino acids in Simmonds. Leucine (10.29%) and cysteine (1.35%) were the most and least abundant amino acids, respectively, in the six avocado chloroplast genomes (Fig 2).

### Repeat sequence analysis

**Fig 3:**
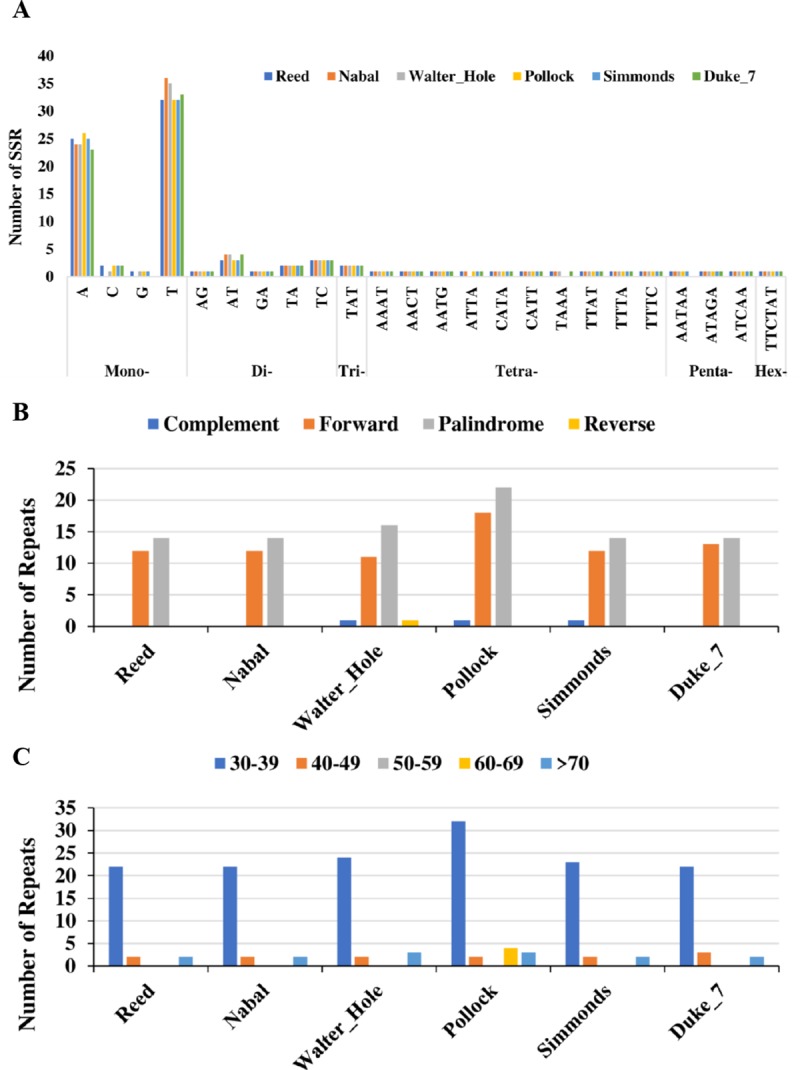
Analysis of repeated sequences in six avocado chloroplast genomes. (A) Frequency of selected motifs of simple sequence repeats (SSRs). (B) Type of repeats in six avocado accessions. (C) Frequency of the repeat sequences longer than 30 bp.

A total of 515 simple sequence repeat (SSR) loci were identified in the six avocado chloroplast genomes (Fig 3A, S2 Table), with each avocado accession containing 84–87 SSRs (mean: 86 SSRs). Among the SSRs, the mono-nucleotide repeat was the most common, accounting for approximately 69.90% of all SSRs, followed by di-nucleotide (12.23%), tetra-nucleotide (11.07%), penta-nucleotide (3.30%), and tri-nucleotide (2.33%) repeats. Hexa-nucleotide repeats (1.17%) were very rare in the six avocado chloroplast genomes. Mono-nucleotide SSRs were especially rich in A/T repeats (approximately 96.39% of all mono-nucleotide SSRs) across the six avocado chloroplast genomes. The SSRs were more abundant in non-coding regions (84.85%) than in protein-coding regions (15.15%) (S2 Table). The protein-coding regions with SSRs included *matK*, *rpoC2*, *cemA*, *petB*, *rps4*, *rpl20*, *rpl22*, *ycf1*, and *ycf2*. The number and distribution of four other repeat types in the six avocado chloroplast genomes were similar and conserved (Fig 3B, S3 Table). The 176 identified repeats belonging to the four other types represented complementary (3), forward (78), palindromic (94), and reverse (1) repeats. Regarding these four other repeat types, Pollock had more (41) than the other five avocado accessions (26–29) (Fig 3B). Most of these repeats were forward and palindromic types that were 30–39 bp long (Fig 3C). These repeat sequences were primarily located in non-coding regions, but a few were distributed in protein-coding regions (*trnS-GCU*, *trnS-GGA*, *trnL-UAG*, *trnT-GCC*, *trnT-GGU*, *trnR-CCU*, *trnS-GGA*, *trnL-UAG*, *psaA*, *psaB*, *clpP*, *rpl2*, *rps14*, *rrn16*, *ccsA*, *ycf1*, and *ycf2*) (S3 Table).

### Inverted repeat contraction and expansion

**Fig 4:**
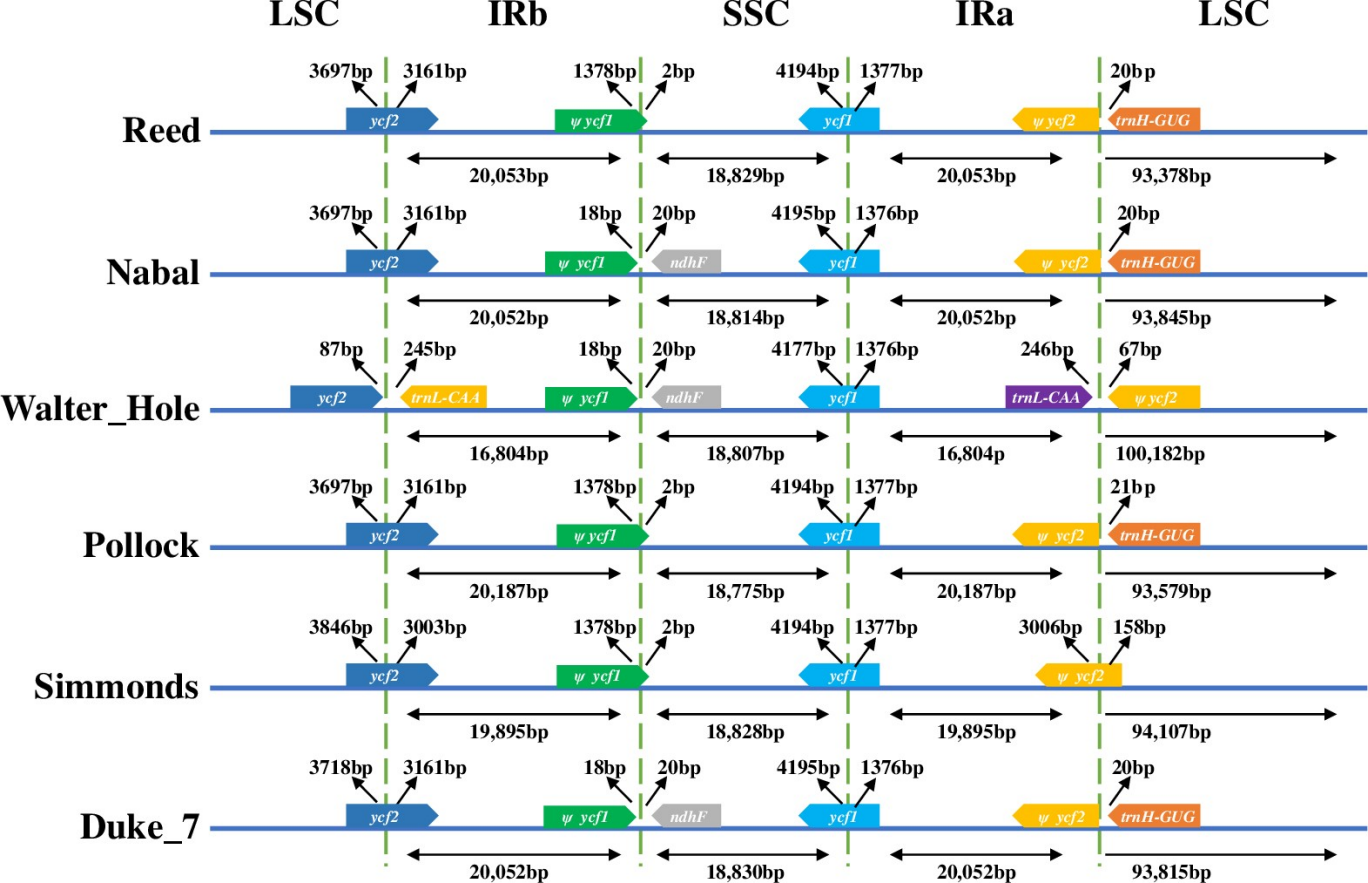
Comparison of the border positions of the large single copy (LSC), small singlecopy (SSC), and inverted repeat (IR) regions in the chloroplast genomes of six avocado accessions. *Ψ* indicates a pseudogene.

The variability in the border structures of six avocado chloroplast genomes was analyzed, and details regarding the LSC, SSC, and IR regions are presented in Fig 4. The *ycf2* gene located in the LSC region extended into the IRb region by 3,005 bp (Simmonds) or 3,163 bp (Duke 7, Nabal, Reed, and Pollock), whereas *ycf2* and *trnL-CAA* were located on either side of the LSC/IRb border, separated by 330 bp in Walter Hole. Like LSC/IRb boundary regions, the SSC/IRa regions also varied. The *ycf1* genes of the six avocado chloroplast genomes were located at the junction of the SSC/IRaregions, and consisted of 5,553 bp (Walter Hole) or 5,571 bp (Duke 7, Nabal, Reed, Pollock, and Simmonds). The *ycf1* pseudogene and *ndhF* were located on either side of the IRb/SSC border and were separated by 38 bp (Nabal, Water Hole, and Duke 7), whereas the *ycf1* pseudogene overlapped the IRb/SSC border in Reed, Pollock, and Simmonds, with 1,378 bp located in the IRb region and 2 bp in the SSC region. Similarly, the *ycf2* pseudogene and *trnH-GUG* were located on either side of the IRa/LSC border and were separated by 20 bp (Reed, Nabal, and Duke 7) and 21 bp (Pollock), whereas *trnL-CAA* and the *ycf2* pseudogene were located 313 bp apart and on either side of the IRa/LSC border (Walter Hole). The *ycf2* pseudogene spanned the IRa/LSC region, with 3,006 bp located in the IRa region and 158 bp in the LSC region.

### Mutation hotspots of chloroplast genomes

**Fig 5:**
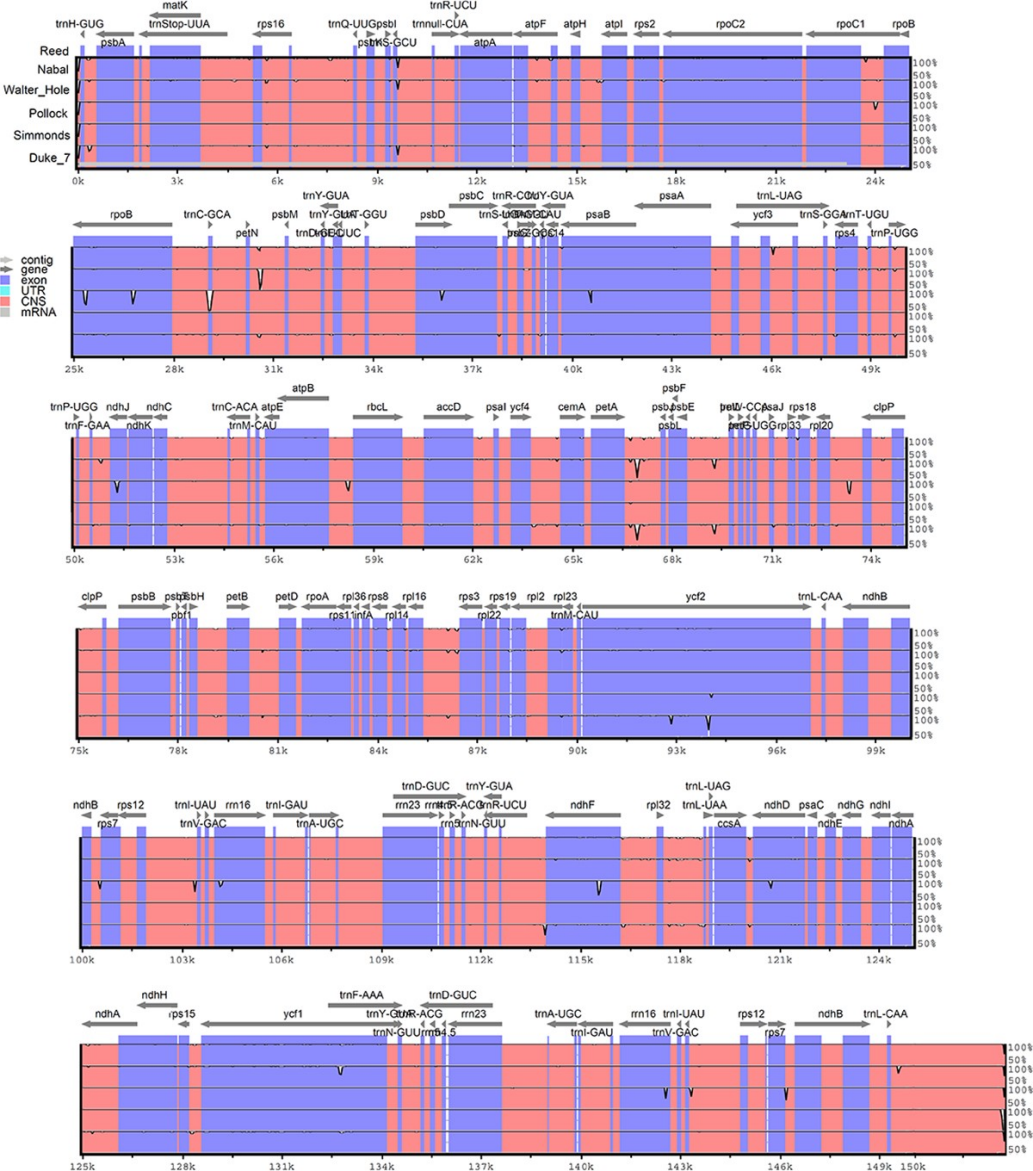
Sequence alignment of chloroplast genomes from six avocado accessions. The sequences were aligned and compared with the mVISTA program. The x-axis presents the coordinates within the chloroplast genome, whereas the y-axis presents the percentage identity, ranging from 50% to 100%. The gray arrows indicate the direction of the gene. Purple bars represent exons, and orange bars represent conserved non-coding sequences.

To determine the extent of the sequence divergence, the six avocado chloroplast genomes were compared with mVISTA (Fig 5). The comparison indicated that the non-coding regions were more divergent than the protein-coding regions, and the LSC and SSC regions included more hypervariable regions than the IR regions (S4 and S5 Tables). One distinct difference between Pollock and the other five avocado accessions was detected in the *trnC-GCA-petN* sequence of the LSC region. Additionally, a distinct difference between Walter Hole and the other five avocado accessions was detected in the intergenic spacer (*petN-psbM*) in the LSC region. Moreover, the *petA-psbJ* sequence in the LSC region of two Mexican races (Walter Hole and Duke 7) was highly divergent from the corresponding sequence in the four Guatemalan and West Indian races.

**Fig 6:**
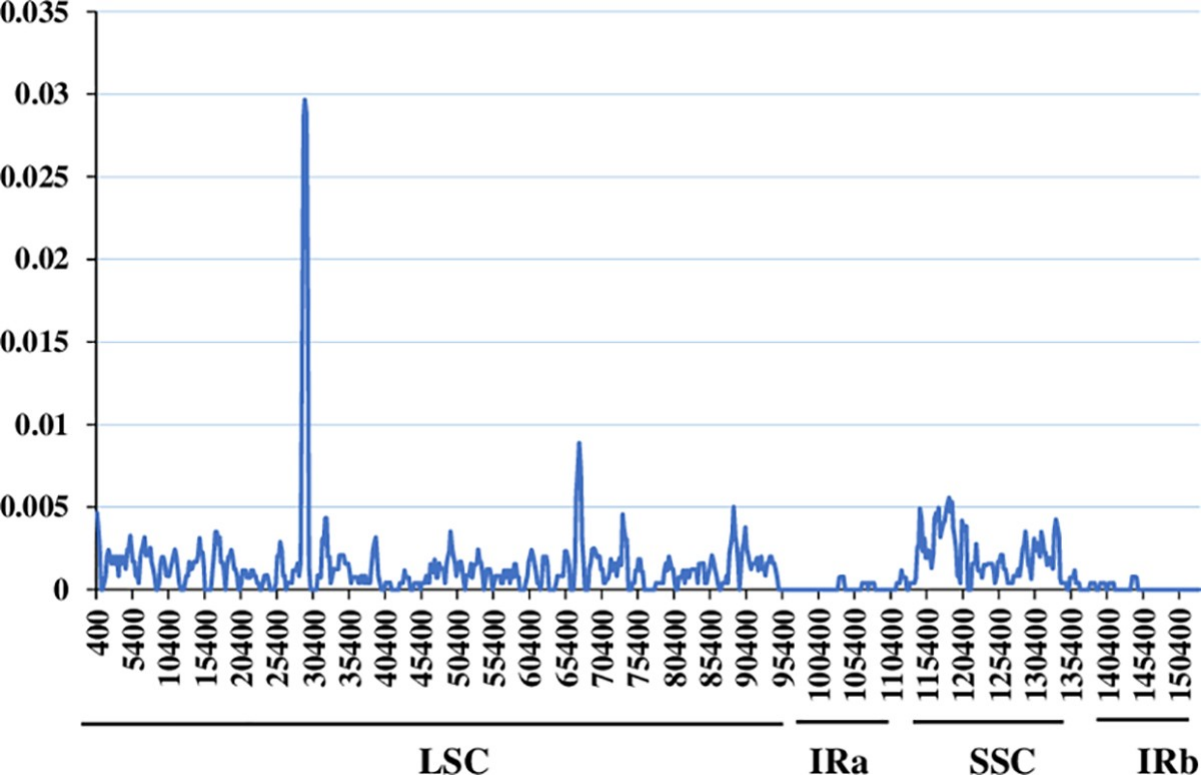
Sliding window analysis of the nucleotide variability (Pi) among six avocado chloroplast genomes. Window length: 800 bp; step size: 200 bp.

A sliding window analysis with DnaSP detected highly variable regions in the six avocado chloroplast genomes. The nucleotide variability (Pi) was calculated to quantify the divergence at the sequence level among the six avocado chloroplast genomes (Fig 6). The Pi value ranged from 0 to 0.0299, with a mean of 0.0012. As expected, the IR regions were more conserved than the LSC and SSC regions. The most divergent region, *trnC-GCA-petN* in the LSC region, had a Pi value of 0.0299, and the *petA-psbJ* sequence in the LSC region had a higher degree of nucleotide variability, with a Pi value of 0.0095.

#### AMOVA analysis

The AMOVA based on 484 SNPs from six avocado chloroplast genomes from three ecological races revealed a clear population separation. When the three populations were considered (Mexican, Guatemalan, and West Indian race subpopulation), 45% of the variation occurred among populations. The three pairwise comparisons of *F*_st_ were significant (*p*<0.001). The closest subpopulations were Guatemalan and West Indian race (*F*_st_ = 00.34, *p*<0.001).Similarly, the Guatemalan subpopulation had almost the same distant from the Mexican subpopulations (*F*_st_ = 00.35, *p*<0.001). The farthest subpopulations were Mexican and West Indian race (*F*_st_ = 00.60, *p*<0.001).

### Phylogenetic analysis

**Fig 7:**
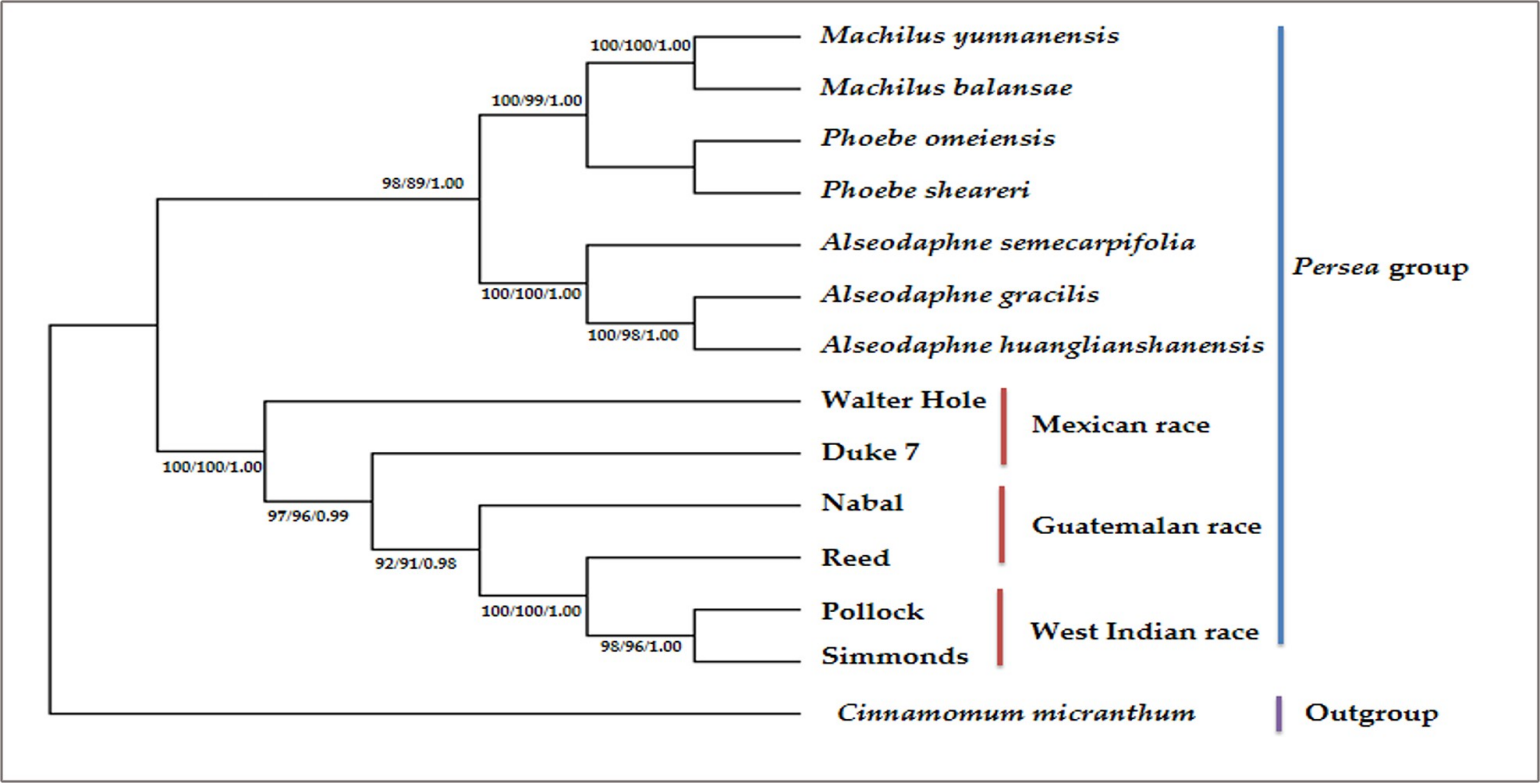
Phylogenetic tree including six avocado accessions based on maximum likelihood (ML), maximum parsimony (MP), and Bayesian inference (BI) methods and 79 common protein-coding genes in the chloroplast genomes. Numbers above the lines represent ML/MP bootstrap values/BI posterior probability.

The intraspecific relationships among six avocado accessions from three ecological races were classified, and eight species from four genera of Lauraceae were set as an outgroup. The dataset for 79 common protein-coding genes in the 14 chloroplast genomes was used to construct phylogenetic trees based on the maximum likelihood (ML), maximum parsimony (MP), and Bayesian Inference (BI) strategies, with different partitioning approaches (Fig 7). The resulting phylogenetic trees had highly similar topologies. Additionally, the six avocado accessions from three ecological races formed a single clade, with high bootstrap and BI support values. Of these six avocado accessions, Walter Hole and Duke 7, classified as *P*. *americana* var. *drymifolia*, were initially clustered in the clade with Nabal and Reed *(P*. *americana* var. *guatemalensis)*, but were then grouped with Pollock and Simmonds (*P*. *americana* var. *americana)*. Pollock and Simmonds had a paraphyletic relationship. Walter Hole was located at the basal position of these six avocado accessions. Additionally, the monophyly of seven samples from the genera *Phoebe*, *Machilus*, and *Alseodaphne* strongly suggested these species were sister species of *P*. *americana*.

## Discussion

The number of sequenced angiosperm chloroplast genomes has increased with the application of next-generation sequencing technology. However, the chloroplast genome sequence of only one species belonging to the genus *Persea* has been reported [44]. In the present study, the six analyzed chloroplast genomes from three avocado ecological races comprised 152,597–152,763 bp, including 79 protein-coding genes within a quadripartite structure (LSC: 93,579–100,182 bp; SSC:18,775–18,830 bp; IR:16,804–20,187 bp). The six avocado (*P*. *americana*) chloroplast genomes consisting of 152,597–152,763 bp were smaller than the published chloroplast genomes of the genera *Phoebe* (152,855 and 152,876 bp) [45] and *Alseodaphne* (153,051–153,099 bp) [46], but were larger than the chloroplast genomes of the genus *Machilus* (152,622 and 152,721 bp) [47], with the exception of Walter Hole (152,597 bp). No clear differences in the chloroplast genome sequence lengths and structures were detected among the three examined avocado ecological races. The six avocado chloroplast genomes analyzed in this study had an overall GC content of approximately 39.11%, similar to the previously published chloroplast genomes of the genera *Phoebe*, *Alseodaphne*, and *Machilus* [45–47]. The GC contents of the LSC and SSC regions were considerably lower than that of the IR regions. This finding is consistent with most of the previously published angiosperm chloroplast genomes, and may have been due to a decrease in the number of AT nucleotides in the rRNA genes [20,48].

The pseudogenes in the chloroplast genome are functionless genes that do not encode a protein; however, pseudogenes may maintain certain gene regulatory functions, with important physiological effects [49–51]. In this study, *ycf1* and *ycf2* sequences encoding proteins with unknown functions in the six avocado chloroplast genomes were identified as pseudogenes. The *ycf1* and *ycf2* genes are common in angiosperm chloroplast genomes, and often become pseudogenes [22,25,48,52,53].The *ycf1*and *ycf2* genes contributed to some of the IRb/SSC and IRa/LSC border structure variations, respectively, in all six avocado chloroplast genomes included in this study. The *ycf1* gene was also partly responsible for the structural variation in the junction between IRb and SSC in the genera *Ziziphus* [22], *Gentiana* [25], *Aconitum* [48], *Lancea* [52], *Papaver* [53], *Camellia* [54], and *Viola* [55]. Compared with the *ycf1* gene in the current study, the *ycf1* gene in the chloroplast genome of most angiosperms is usually larger and more diverse. Thus, it is often partially duplicated in the IRb region [20,22,25,53], which may result in a loss of protein-coding ability and variation in the IRb/SSC border structure.

Previous studies confirmed that repeat sequences are vital for the rearrangement and stabilization of chloroplast genomes [56]. In the current study, palindromic, forward, complementary, and reverse repeats were detected in the six avocado chloroplast genomes, with substantially more palindromic and forward repeats than complementary and reverse repeats. These four repeat types represented 97.73% of the total number of repeats. Additionally, these four types of repeats were more abundant in Pollock than in the other five avocado accessions. Similarly, palindromic and forward repeats represent the main repeat type in other plant species, including those in the genera *Ziziphus* [22], *Lancea* [52], and *Papaver* [53]. Most of these four repeat types were distributed in the intron regions and intergenic spacers, similar to findings for other angiosperms [20,22,25,45–48,53]. In the current study, 515 SSR loci were detected among the six avocado chloroplast genomes, and the most common repeat comprised mononucleotides (A/T) (67.38%).This percentage was slightly higher than that (60.70%) of the transcriptomes from three avocado ecological races in our previous study [19]. The bias towards A/T in this study may have been due to the remnants of poly-A tails. Moreover, similar to the distribution of palindromic, forward, complementary, and reverse repeats, most SSRs were also identified in non-coding regions, and only a small proportion was detected in protein-coding regions, which is in agreement with the results for other angiosperms [20,22,25,45–48,53]. This considerable abundance of repeat elements and SSR loci possibly contributes to the chloroplast genome size variation and divergence [57].

A DNA barcode refers to representative, standard, mutated, easily amplified, and relatively short fragments of DNA in an organism. Such a bar code may be useful for distinguishing a species within a given taxonomic group and is considered to be an effective molecular tool for the assignment of plant species [25,58]. Therefore, more reliable and effective DNA barcodes with high mutation rates should be mined for assigning races, investigating genetic diversity, and certifying avocado germplasm varieties. The complete chloroplast genome has a relatively conserved sequence from 110,000 to 160,000 bp, which far exceeds the length of conventional molecular markers and offers more variable loci to differentiate closely related species [25,58]. In our study, the sequences of six chloroplast genomes from three avocado ecological races were highly similar, with only a few regions that were highly mutated. These results imply the chloroplast genomes of the three investigated avocado ecological races are highly conserved. An analysis of the LSC region with mVISTA and DnaSP revealed three major mutation hotspots (*trnC-GCA-petN*, *petN-psbM*, and *petA-psbJ*) in the six avocado chloroplast genomes. These regions may include highly variable DNA barcodes useful for identifying avocado germplasms. They may also provide sufficient genetic markers for illuminating the phylogeny of the three avocado ecological races. Specifically, the *petA-psbJ* sequence of the Mexican race is highly divergent from that of the Guatemalan and West Indian races. To the best of our knowledge, relatively few useful race-specific markers have been detected in avocado [18]. Mexican race-specific markers may be identified in the *petA-psbJ* region, and will help to validate the racial origin of avocado accessions with an undetermined race.

The *Persea* group belongs to the family Lauraceae of the order Laurales, and includes the genera *Persea*, *Machilus*, *Alseodaphne*, *Phoebe*, *Nothaphoebe*, and *Caryodaphnopsis* [59]. Most of the members of the *Persea* group exhibit a tropical and subtropical amphi-Pacific disjunct distribution [59]. The strongly supported phylogeny of *Persea* and its allied genera was clarified in previous studies based on chloroplast genomic data [44,45,59–61]. Moreover, the divergence of *P*. *americana* occurred approximately 10 million years ago [59]. However, the phylogenetic relationships among three *P*. *Americana* avocado ecological races have not been established because of a lack of sequenced chloroplast genomes for the three ecological races. To clarify the phylogenetic relationships of three avocado ecological races, six avocado accessions from three ecological races as well as three genera belonging to the *Persea* group and one *Cinnamomum* species were used to construct phylogenetic trees based on ML, MP, and BI methods and 79 common protein-coding genes. The basic topologies were similar in the ML, MP, and BI analyses, implying that the Mexican race is located at the basal position of the *P*. *americana* clade. Additionally, the Mexican and Guatemalan races are more closely related to each other than to the West Indian race. This observation agrees with the results of our previous studies involving specific length amplified fragment sequencing and transcriptomes [18,19]. These six avocado accessions from three ecological races clustered in the same clade, and the other three genera belonging to the *Persea* group formed a sister clade with a high bootstrap value. This result is similar to that of previous phylogenomic analyses [44,45,62].

In conclusion, the results of a phylogenetic analysis based on the chloroplast genomes greatly enhanced our understanding of the evolutionary relationships among three avocado ecological races. In future investigations, additional chloroplast genome datasets are needed to test the phylogenetic relationships of avocado as well as the evolution of avocado races.

## Conclusions

In this study, six complete avocado chloroplast genomes were analyzed and compared regarding sequence variations and molecular evolution. The avocado chloroplast genome forms a representative quadripartite molecular structure, similar to the chloroplast genomes of other angiosperms. Additionally, the 176 repeats detected in the chloroplast genomes comprised complementary (3), forward (78), palindromic (94), and reverse (1) repeat elements. The results of SSR mining indicated the most common SSRs in six complete chloroplast genomes were mono-nucleotides, followed by di-nucleotides, tetra-nucleotides, penta-nucleotides, tri-nucleotides, and hexa-nucleotides. Furthermore, three mutation hotspots in the LSC region (*trnC-GCA-petN*, *petN-psbM*, and *petA-psbJ*) may be useful as DNA barcodes for future studies of avocado population genetics and phylogenetic relationships. A phylogenetic analysis based on protein-coding genes revealed that the six avocado accessions from three ecological races formed a highly supported monophyletic clade. The six chloroplast genomes provide important biological information for further discriminating between avocado races and for elucidating the phylogenetic relationships associated with avocado.

## Supporting information

S1 Table Genes present in six avocado chloroplast genomes. (XLSX)

S2 Table Simple sequence repeats in six avocado chloroplast genomes. (XLSX)

S3 Table Distribution of repeat sequences in six avocado chloroplast genomes. (XLSX)

S4 Table Percentages of variable characteristics in protein-coding and non-coding regions in six avocado chloroplast genomes. (XLSX)

S5 Table Single nucleotide polymorphisms (SNPs) in six avocado chloroplast genome. (XLSX)
